# Integrated multisource estimates of mortality for Thailand in 2005

**DOI:** 10.1186/1478-7954-8-10

**Published:** 2010-05-18

**Authors:** Peter Byass

**Affiliations:** 1 Department of Public Health and Clinical Medicine, Umeå Centre for Global Health Research, Umeå University, Sweden

## Abstract

Estimates of mortality in Thailand during 2005 have been published, integrating multiple data sources including national vital registration and a national follow-up cluster sample, covering both deaths in health facilities (approximately one-third) and elsewhere. The methodological challenge is to make the best use of the existing data, supplemented by additional data that are feasible to obtain, in order to arrive at the best possible overall estimates of mortality. In this case, information from the national vital registration database was supplemented by a verbal autopsy survey of approximately 2.5% of deaths, the latter being used to validate routine cause-of-death data and information from medical records. This led to a revised national cause-specific mortality envelope for Thailand in 2005, amounting to 447,104 deaths. However, difficulties over standardizing verbal autopsy interpretation may mean that there are still some uncertainties in these revised estimates.

## Introduction

**Figure 1:**
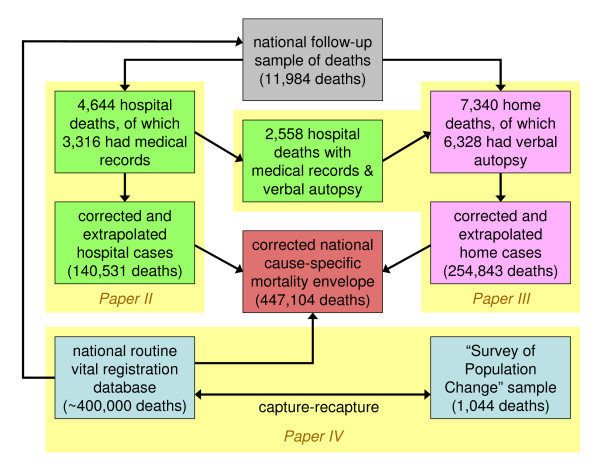
**Conceptual framework showing relationships between the multiple data sources involved in estimating mortality in Thailand during 2005**.

This commentary relates to a set of four papers by Rao and colleagues that relate to a detailed investigation of cause-specific mortality in Thailand during 2005, integrating a number of different data sources. Paper I considers the rationale and methods for starting with routine death registration data, but complementing these with other data in order to arrive at more coherent overall estimates [1]. About one-third of registered deaths in Thailand occur in hospitals, and paper II looks into the validity of the registered causes of these deaths, using additional information from medical records and verbal autopsies, in order to attribute misclassification errors [2]. For the majority of deaths, which occur outside hospitals, registered causes can only be validated by carrying out verbal autopsies, and this process is covered in paper III [3], which also provides part of the validation comparisons for the hospital deaths in paper II. Paper IV then integrates the findings from papers II and III into a complete overview and estimate of mortality for Thailand in 2005, with a discussion of implications for practice and policy [4]. The somewhat complex inter-relationships between these four papers are illustrated conceptually in Figure 1, with paper I being an overview of the whole.

### The context of Thailand

Although paper I characterizes Thailand in terms of its record in documenting mortality in recent decades, it is also important to understand the position of Thailand in relation to other nations in terms of demographic, socioeconomic, and other contextual factors. The approach used in this whole series of papers is not uniquely applicable to Thailand, but at the same time would not be appropriate in appreciably more or less developed contexts.

According to UNICEF [5], in 2005, Thailand was ranked 108 out of 190 of the world's countries in terms of under-5 mortality, putting it close to countries such as Saudi Arabia, Tunisia, Colombia, Jamaica, and Romania. Cumulative under-5 mortality was 21 per 1,000 live births, with a gross national income per capita of US$ 2,750 and life expectancy at birth of 71 years. These parameters are broadly comparable with other countries in the East Asia and Pacific region, as well as being similar to overall figures for countries in Latin America and the Caribbean and the countries of Central and Eastern Europe and the Commonwealth of Independent States. Thailand's status in these terms is substantially ahead of UNICEF's "developing countries" group but substantially behind the "industrialized countries" group.

Mathers and colleagues in 2005 [6] characterized Thailand as being among a group of 28 mainly middle-income countries that were supplying cause-of-death information to WHO but whose data were judged to be of low quality. A national study of mortality statistics in Thailand [7] identified a number of obstacles to effective routine cause-of-death registration, noting problems both among the one-third of deaths occurring at health facilities and the two-thirds occurring outside.

## Discussion

### Methodological reflections

A large and complex study of this kind inevitably raises a number of methodological challenges, several of which are worth noting. The basic challenge is to make the best use of existing data (in this case, the national routine vital registration database), while also recognizing inherent limitations and devising strategies for obtaining supplementary data that can address some of those limitations. Any such supplementary data must be realistically obtainable, as was the case here for the national cluster sample verbal autopsy study undertaken for approximately 2.5% of deaths. Sample size determination for studies of cause-specific mortality is not straightforward, with an obvious dependence on the distribution of particular causes.

Paper I describes the approach used here, using assumptions centered on the magnitude of the 21st ranked cause of death in the population, and this is a helpful example for other studies. Paper I, in considering the verbal autopsies (VA) to be undertaken in the national follow-up sample, also helpfully stresses the general situation that there exists no "gold standard" for comparing VA cause-of-death data with routine registration, or for that matter medical records data, with the consequence that kappa measures of agreement are the method of choice.

Papers II and III are both concerned with validating samples of registered causes of death. Paper II focuses on determining cause of death from abstracted medical records and comparing these with registered causes. This is not an easy process, since in many cases, it is likely that at least part of the pre-mortem medical records will have been written by the same practitioner who assigned the post-mortem cause of death for registration, and so there is a risk of circularity between matters of diagnosis and cause of death. The process used here tried to allocate more weight to histopathology, laboratory, and imaging evidence from the medical record, which may have offset these difficulties to some extent. In principle (even if not possible in practice), it would have been interesting to compare medical records cases diagnosed and subsequently certified by the same practitioner with those involving different practitioners. In addition, this part of the overall validation makes creative use of the subset of hospital deaths that also had medical records and were included in the verbal autopsy follow-up process described in paper III.

Paper III is intriguing in that VA is used not because of a lack of routine registration, as is usually the case, but in order to provide a framework to validate the routine cause-of-death data. Unfortunately for studies of this kind, VA methods (interview protocols, interpretation of interview material, analytical approaches to findings) are by no means standardized or unchanging [8]. Here, the research group decided to use a single physician to interpret each VA questionnaire, with the possibility of seeking a second opinion. One assumes that the general practitioners involved were likely to have studied medicine in Thailand and be familiar with local clinical practice. It seems that the research group was determined to achieve a single cause of death per case, since if consensus between the original and second opinions could not be achieved, an ill-defined cause was assigned. This seems to represent an unnecessary loss of information, rather than the alternative course of recording the individual opinions. No data are presented as to how many individual physician coders were involved or the extent of inter-observer variation in their conclusions, which is a pity. Then, a central team further reviewed the cause-of-death data and, if necessary, overruled initial decisions, although it is not clear how often or in what circumstances this occurred. This entire process, even if performed fastidiously, leaves a lot of room for subjectivity and undesirable variations.

The issue of the overall validity of the VA interpretation process, given that there is no objective "gold standard," is critical to the integrity of the whole study. Figure 1 shows that not only was the VA process crucial to the interpretation of deaths outside health facilities, but the critical cross-validating link (relating to the 2,558 hospital deaths that also had medical records and VA data) is also dependent on the validity of the VA process as implemented here.

Paper IV introduces the important additional concept of the capture-recapture approach in order to assess the magnitude of what has not been measured. This is a key component in making effective use of substantial existing data (in this case, the national vital registration database) even where there may be doubts as to completeness.

### Public health implications and generalizability

As I have discussed previously [9], the choice of appropriate approaches to cause-of-death data depends very much on the target audience. Here, the overall aim appears to be the characterization with increased reliability and precision of the national cause-specific mortality situation in Thailand. One assumes, therefore, that this is targeted toward the national level in Thailand, as well as for upward reporting to the international agencies. This has to be coupled with the context of Thailand, a middle-income country in which an appreciable proportion of deaths occur in health facilities, in order to understand that the study constitutes a good example of how to arrive at a reliable national picture of causes of death in a middle-income country, maximizing the utility of various data sources.

Nevertheless, the overall process of arriving at these estimates was quite involved, and also presumably time-consuming and expensive. The mere fact that 2010 sees the publication of estimates for 2005 is perhaps indicative of this complexity, but also to some extent depreciates the public health value of the results. Whether the approach used is something that could be streamlined and turned around faster, as part of more routine rather than research processes, is a matter for further consideration. It is also important to recognize that this approach possibly has limited value to downstream levels, such as provincial directors of public health, who probably need simpler and more rapid assessments of cause-specific mortality in their local areas.

All of this work is predicated on the conventional medical model of assigning a single cause of death to each case, utilizing no measure of uncertainty as to cause at the individual case level. Whether that is the approach that maximizes the public health value of mortality data is a wide-ranging issue, beyond the scope of this commentary. However, there are signs within the data presented that suggest allowing multiple, proportional causes per case might be helpful (for example, signs of crossover between HIV/AIDS and pulmonary tuberculosis as determined by medical records and VA in paper III, table six) [3].

Another research group also involving Rao has recently published similar work from nearby Vietnam for 2006 [10]. Different methods were applied to VA interpretation, with VA questionnaires "reviewed by a team of experienced medical doctors at each (regional) medical university." Interestingly, there are both appreciable similarities and differences in the cause-of-death distributions reported, and one has to ask the question as to which of these similarities and differences are due to variations in local understandings and methods, as opposed to genuine differences in causes of death. This is a crucial issue for any generalized understanding of public health involving international comparisons. For example, in Vietnam, the second-leading cause of mortality in the under-15 age group was pneumonia, which did not appear in the leading causes for that age group in Thailand (paper IV, table two) [4]. This is an example of a major difference that is not easy to explain.

These difficulties suggest that there is substantial need for further work in developing cause-of-death methods, particularly in the area of probabilistic modeling as a means of VA interpretation on a standardized basis [8]. It would be extremely interesting to run both the Thai and Vietnamese VA data through the same probabilistic model to see how the cause-of-death differences between the two countries compare with the differences as determined by physician review. This would be a particularly interesting comparison between these two countries, which have some regional and socioeconomic similarities but significant differences in their traditions of medicine and medical education.

## Conclusions

These four papers represent an extremely interesting insight into mortality patterns in Thailand as determined in a comprehensive study that integrated multiple data sources. Nevertheless, issues of standardization, particularly with respect to some of the methodological components, leave unanswered questions about the overall conclusions, particularly in terms of international comparison. In terms of conclusions on the overall process of undertaking these multisource revisionary estimates of the routine death registration process, one has to ask the fundamental question as to whether doing so could lead to substantially different public health policies and practice. Figures one and two in paper IV give a resounding "yes" to that question [4], and the methods and analyses as described in this set of papers suggest that the revised mortality envelope estimate is more credible than the underlying routine registration - even if it still does not necessarily represent absolute "truth." The authors quite rightly observe in paper IV that their approach is not a long-term substitute for more complete and accurate vital registration processes in countries such as Thailand, but it may nevertheless offer medium-term benefits and stimulate the development of better registration strategies on a sustainable basis.

## Competing interests

The author declares that he has no competing interests.
